# STK3 promotes gastric carcinogenesis by activating Ras-MAPK mediated cell cycle progression and serves as an independent prognostic biomarker

**DOI:** 10.1186/s12943-021-01451-2

**Published:** 2021-11-12

**Authors:** Bonan Chen, Wai Nok Chan, Chun Wai Mui, Xiaoli Liu, Jinglin Zhang, Yifei Wang, Alvin H. K. Cheung, Aden K. Y. Chan, Ronald C. K. Chan, Kam Tong Leung, Yujuan Dong, Yi Pan, Huixing Ke, Li Liang, Zhaocai Zhou, Chi Chun Wong, William K. K. Wu, Alfred S. L. Cheng, Jun Yu, Kwok Wai Lo, Ka Fai To, Wei Kang

**Affiliations:** 1 Department of Anatomical and Cellular Pathology, State Key Laboratory of Translational Oncology, Prince of Wales Hospital, The Chinese University of Hong Kong, Shatin, Hong Kong, SAR People’s Republic of China; 2 Institute of Digestive Disease, State Key Laboratory of Digestive Disease, The Chinese University of Hong Kong, Hong Kong, SAR People’s Republic of China; 3 Li Ka Shing Institute of Health Science, Sir Y.K. Pao Cancer Center, The Chinese University of Hong Kong, Hong Kong, SAR People’s Republic of China; 4 Department of Pediatrics, The Chinese University of Hong Kong, Hong Kong, SAR People’s Republic of China; 5 Department of Pathology, National Cancer Center, National Clinical Research Center for Cancer, Cancer Hospital, Chinese Academy of Medical Sciences and Peking Union Medical College, Beijing, People’s Republic of China; 6 Department of Respiratory and Critical Care Medicine, China National Center of Gerontology, Bejing Hospital, Beijing, People’s Republic of China; 7 Department of Pathology, Nanfang Hospital and Basic Medical College, Southern Medical University, Guangdong Province Key Laboratory of Molecular Tumor Pathology, Guangzhou, People’s Republic of China; 8 State Key Laboratory of Genetic Engineering, School of Life Sciences, Zhongshan Hospital, Fudan University, Shanghai, People’s Republic of China; 9 Department of Anaesthesia and Intensive Care, The Chinese University of Hong Kong, Hong Kong, SAR People’s Republic of China; 10 School of Biomedical Sciences, The Chinese University of Hong Kong, Hong Kong, SAR People’s Republic of China; 11 Department of Medicine and Therapeutics, The Chinese University of Hong Kong, Hong Kong, SAR People’s Republic of China

**Keywords:** STK3, Gastric cancer, Oncogene, Ras-MAPK signaling

## Main text

Gastric cancer (GC) has long been a major cancer burden. In 2020, it is responsible for 1,089,103 new cases and 768,793 deaths globally, with more than 656,349 new cases and 435,211 deaths from Eastern Asia [1]. Notably, the Hippo pathway shows the critical tumor-suppressor function and is frequently dysregulated in GC [2]. The protein STK3/MST2 is a serine/threonine-protein kinase, a homologue of the Hippo protein in *Drosophila*, which plays an essential role in the Hippo signaling pathway. Traditionally, the activated STK3 kinase will undergo dimerization and negatively regulate the yes-associated protein 1 (YAP1) and WW Domain-Containing Transcription Regulator Protein 1 (TAZ), which trigger the expression of proliferation genes and inhibit apoptosis [3]. So, STK3 was proposed to exert anti-cancer functions in tumors.

**Fig. 1:**
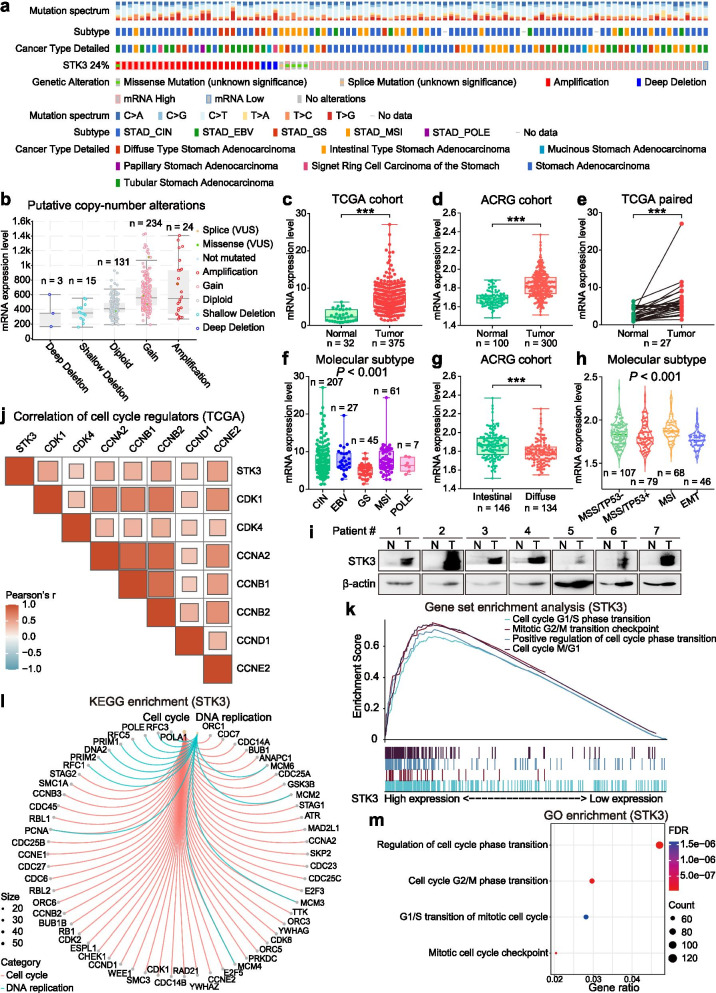
STK3 is upregulated in GC patients (***, *P* < 0.001). **a** The STK3 genetic alterations (gene amplification, deep deletion, or somatic mutation) and mRNA expression in primary GC samples from the TCGA cohort (total alteration rate: 24%). **b** The positive correlation of STK3 copy-number alterations with its mRNA expression. **c, d** STK3 mRNA expression is upregulated in the tumor tissues compared with it in normal tissue group from TCGA and ACRG cohorts. **e** STK3 is upregulated in tumor samples compared with paired adjacent nontumorous samples. **f** The STK3 mRNA is differently expressed across five molecular subtypes in TCGA cohort. STK3 was significantly upregulated in EBV-positive and microsatellite-instable types of GC (*P* < 0.001). **g** STK3 mRNA expression is abundantly expressed in intestinal-type GCs compared with diffuse-type GCs. **h** The STK3 mRNA expression in four molecular subtypes proposed by ACRG cohort (*P* < 0.001). **i** The STK3 protein expression is increased in GC samples compared with the paired normal controls. **j** The expression of STK3 is positively correlated with cell cycle genes from TCGA cohort. **k** The cell cycle-related pathways are positively correlated with high STK3 expression by GSEA (*P* < 0.05). **l** STK3-related DEGs (|Fold Change| > 2, *P* < 0.05) were enriched in cell cycle and DNA replication through KEGG enrichment analysis (*FDR* < 0.001). **m** STK3-related DEGs were enriched in cell cycle progression through GO enrichment analysis (*FDR* < 0.001)

However, we found that the amplification and copy number gain of STK3 is a common event in GC cases from The Cancer Genome Atlas (TCGA) cohort (Fig. 1a and b). Additionally, STK3 mRNA level was upregulated in tumor samples compared with it in normal tissues from both TCGA and Asian Cancer Research Group (ACRG) cohorts (Fig. 1c and d). Simultaneously, when tumorous samples were paired up with adjacent nontumorous tissues, the mRNA expression of STK3 was generally elevated (Fig. 1e). Across the five molecular subtypes defined by TCGA, STK3 expression was most upregulated in EBV-positive and microsatellite-instable types of GC (Fig. 1f). Meanwhile, STK3 was abundantly expressed in the intestinal-type (Fig. 1g) and the microsatellite-instable subtype of GC cases (Fig. 1h) from the ACRG cohort. The STK3 protein levels demonstrated increased expression in cancer samples compared with the paired normal epithelium samples from Hong Kong GC cohort (Fig. 1i). Likewise, in most of the solid tumors, the STK3 exhibited high expression from TCGA cohort (Additional file 1: Fig. S1) [4]. To further investigate the functional role of STK3 in gastric carcinogenesis, we employed bioinformatics to analyze the gene expression profile of 375 GC patients from TCGA cohort. The correlation heatmap demonstrated that STK3 expression was positively correlated with the expression of cell cycle regulators like CDK1, CCNB2 and CCNE2 [5] (Fig. 1j). The gene set enrichment analysis (GSEA) revealed that genes related to cell cycle progression were positively correlated with high STK3 expression (Fig. 1k). Then, differentially expressed genes (DEGs, fold change > 2, *P* < 0.05) were distinguished by comparing the 10% samples with the lowest SKT3 expression (*n* = 37) and the 10% samples with the highest STK3 expression (*n* = 37). KEGG pathway analysis showed that these DEGs were mainly involved in DNA replication and cell cycle progression (Fig. 1l). Similarly, the gene ontology (GO) analysis indicated that DEGs were associated with cell cycle checkpoints and cell cycle arrest (Fig. 1m). The results in this part indicate that the STK3 is highly expressed in GC and might exert oncogenic function. Notably, studies have demonstrated that the expression of STK3 was also increased in other cancer types such as prostate cancer and leukemia, which supported our conclusion [6–8].

**Fig. 2:**
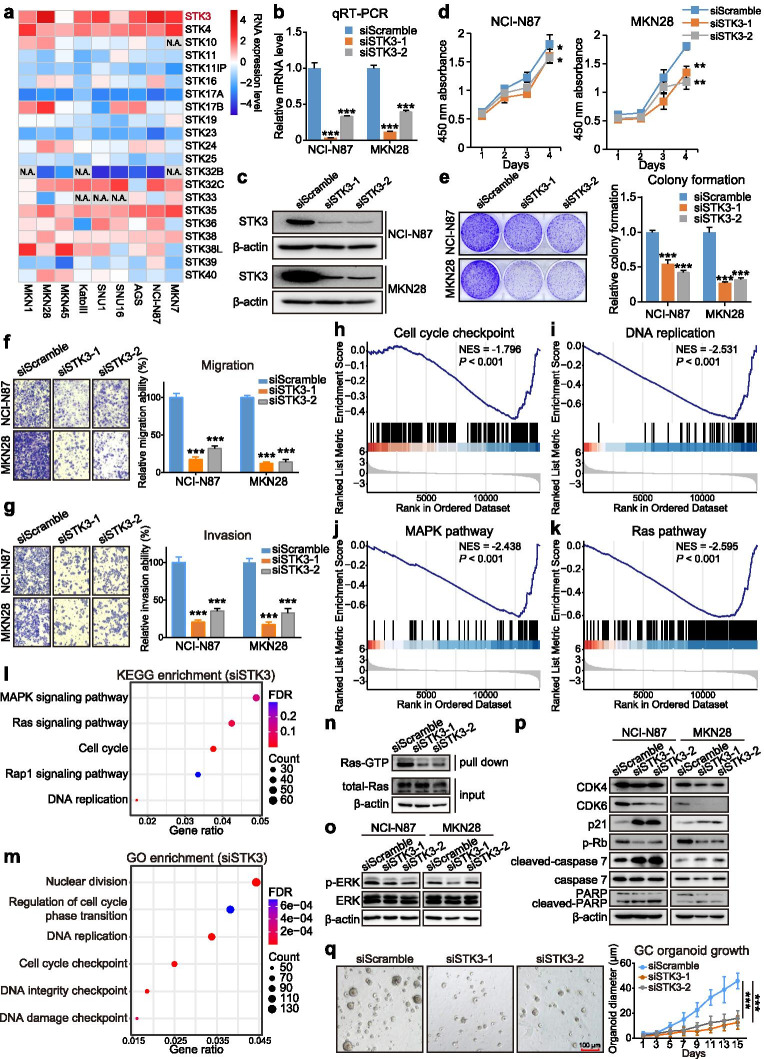
STK3 promotes GC by activating the Ras-MAPK signaling pathway (*, *P* < 0.05; **, *P* < 0.01; ***, *P* < 0.001). **a** The heatmap of STK family members in nine GC cell lines. STK3 is abundantly expressed, especially in NCI-N87 and MKN28 cells. **b, c** The STK3 mRNA and protein expression were significantly decreased after siSTK3 transfection in NCI-N87 and MKN28 cells. **d** STK3 knockdown suppressed GC cell proliferation. **e** Depletion of STK3 inhibited monolayer colony formation of the cancer cells. **f, g** GC cell migration and invasion abilities were impaired with the siSTK3 transfection. **h, i, j, k** GSEA demonstrated that the STK3 depletion was negatively correlated with cell cycle checkpoint, DNA replication, MAPK pathway, and Ras pathway. **l, m** In STK3-depleted cells, the DEGs (|Fold Change| > 1.5, *P* < 0.05) were enriched in the Ras-MAPK signaling pathway, cell cycle, and DNA replication through KEGG and GO enrichment analysis. **n** STK3 knockdown blocked the Ras activation in MKN28 cells. **o** The ERK is dephosphorylated and inactivated in the siSTK3 transfectants. **p** Knocking down STK3 induced G1 phase cell cycle arrest and apoptosis, which were confirmed by the downregulation of CDK4, CDK6, and p-Rb, and activation of cleaved-caspase 7 and cleaved-PARP. **q** STK3 depletion significantly suppressed the GC-derived organoid growth. Scale bar = 100 μm

We then asked if STK3 still possesses oncogenic properties at in vitro level. As expected, STK3 was overexpressed substantially in the nine GC cell lines (Fig. 2a). By siRNA-medicated knockdown, the mRNA expression (Fig. 2b) and protein level (Fig. 2c) of STK3 were decreased. Knocking down STK3 inhibited cell growth, monolayer colony formation, migration, and invasion of GC cells (Fig. 2d, e, f, and g), which indicated that STK3 plays a promoting role in GC. To further investigate the downstream signalings of STK3 in gastric tumorigenesis, we employed GSEA, GO, and KEGG analysis. GSEA revealed that STK3 depletion inhibited cell cycle and DNA replication signatures (Fig. 2h and i). Interestingly, we found that in the siSTK3 transfectants, the MAPK and Ras signaling pathways were significantly suppressed (Fig. 2j and k). Similarly, both KEGG and GO enrichment analysis revealed that the above pathways were affected when STK3 was knocked down (Fig. 2l and m). To further confirm that Ras-MAPK serves as the crucial downstream pathway of STK3 to support tumor development, we performed pull-down assay and Western blot analysis. The Ras-GTP (active Ras form) demonstrated decreased level in STK3 knockdown cells (Fig. 2n). At the same time, the p-ERK was consequently dephosphorylated (Fig. 2o), suggesting STK3 tightly regulates the Ras-MAPK pathway. Multiple studies have confirmed that Ras mediates the activation of MAPK signaling pathway and plays an essential role in carcinogenesis [9, 10], which literally supports STK3 serves as an oncogene in GC. In the STK3-depleted cells, the cell cycle checkpoints such as CDK4 and CDK6 were downregulated, while p21, an inhibitor of cell cycle regulator CDK2, was activated [11]. In addition, the levels of cleaved PARP and caspase 7, both involved in the process of apoptosis, were increased in STK3-depleted cells (Fig. 2p). By functional tests, we also observed that STK3 knockdown significantly suppressed the GC-derived organoid growth (Fig. 2q). All the data support that STK3 promotes gastric carcinogenesis, which is mediated by the Ras-MAPK pathway.

**Fig. 3:**
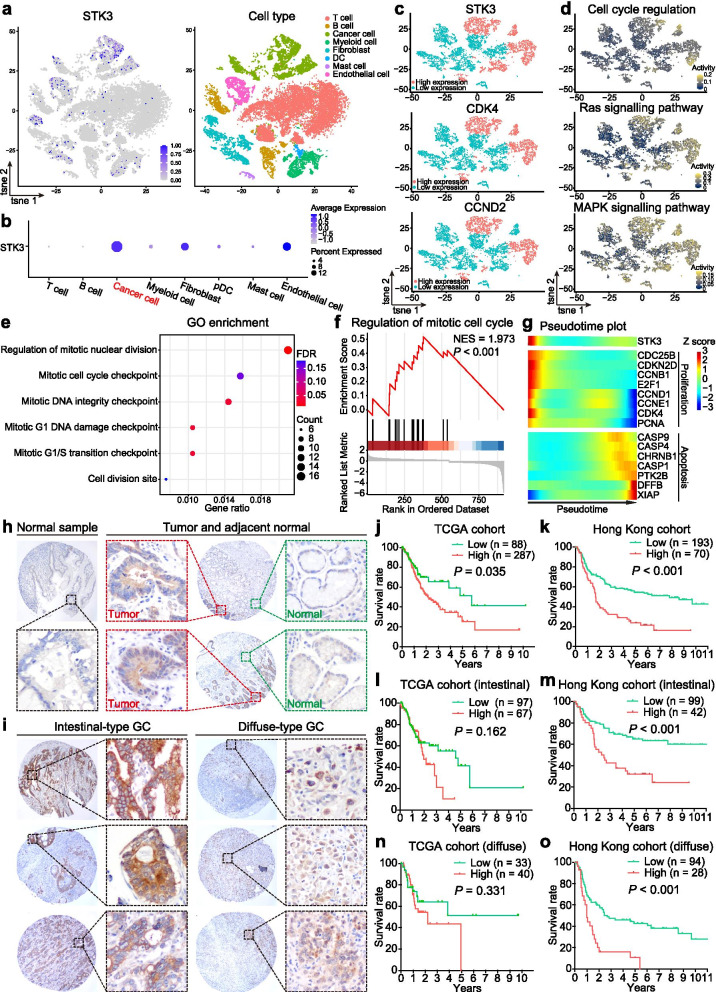
STK3 promotes tumorigenesis by activating the Ras-MAPK pathway and serves as an independent prognostic biomarker. **a, b** STK3 was highly expressed in cancer cells, endothelial cells, and cancer associated fibroblasts. **c** The t-SNE plots of high and low expression populations of STK3, CDK4, and CCND2 in GC single-cell resolution. **d** The activities of cell cycle regulation, Ras signaling pathway, MAPK signaling pathway in GC cells. **e** In single-cell level, DEGs (in rows, q-value < 10^− 10^) between STK3 high and low expression were enriched in cell proliferation-related pathway by GO enrichment analysis. **f** Regulation of mitotic cell cycle was positively correlated with high expression of STK3 through GSEA from single-cell resolution (*P* < 0.001). **g** The proliferation and apoptosis biomarkers along with the functional pseudotime in GC development. STK3 was co-upregulated with proliferation biomarkers in the early state of tumor development. **h** Representative images of IHC staining of STK3 from GC tissue microarray. STK3 was predominantly localized in the cytoplasm of the cancer cells, while it demonstrated negative expression in the adjacent epithelium tissue. **i** STK3 was highly expressed in both intestinal-type and diffuse-type GC samples. **j, k** Overexpressed STK3 was associated with poor disease-specific survival in primary GCs (TCGA cohort, *n* = 375, *P* = 0.035; Hong Kong cohort, *n* = 263, *P* < 0.001). **l, m** STK3 abundance predicted poor disease-specific survival in intestinal-type GC patients (TCGA cohort, *n* = 164, *P* = 0.162; Hong Kong cohort, *n* = 141, *P* < 0.001). **n, o** In diffuse-type GCs, the upregulation of STK3 was associated with unfavorable clinical outcomes (TCGA cohort, *n* = 73, *P* = 0.331; Hong Kong cohort, *n* = 122, *P* < 0.001)

To further reveal the oncogenic role of STK3 and the STK3-related downstream signalings in a spatial and temporal manner, we employed a single-cell RNA sequencing (scRNA-seq) dataset and identified the GC cell population [12]. Surprisingly, we found the STK3 exhibited high expression in cancer cells, endothelial cells, and cancer associated fibroblasts (Fig. 3a and b). The abundance of STK3 in cancer cells and stromal cells consolidates that STK3 is a potent oncogene in GC. Meanwhile, the cancer cell populations with high expression of STK3, CDK4, and CCND2 were almost overlapped, suggesting a positive correlation among these genes in GC development (Fig. 3c). Strikingly, the cell populations with high STK3 expression demonstrated extremely high activities in cell cycle regulation, Ras signaling pathway, and MAPK signaling pathway, reinforcing the linkage between STK3 activation and tumor progression (Fig. 3d). To further support the notion that STK3 promotes cancer cell proliferation and activates oncogenesis pathways, we employed GO enrichment and GSEA to analyze the DEGs between two clusters, STK3 high- and low-expressing GC cells. As shown in Fig. 3e and f, DEGs were enriched in cell division and mitotic regulation, especially in the regulation of the mitotic cell cycle checkpoint (*P* < 0.001). More importantly, in the early state of tumor development, STK3 demonstrated high expression level, and the proliferation-associated genes, such as E2F1, CCND1, CDK4, and PCNA, followed similar kinetic trends with STK3, which was revealed by functional pseudotime analysis from a scRNA-seq dataset (Fig. 3g). However, the early state from the single-cell resolution indicates the tumor cell evolution process instead of the clinicopathological tumor staging. As we know, GC is a highly heterogeneous tumor. In each tumor stage, the cancer cells might be distributed in early, middle or late states in terms of their evolution lifespan. In terms of the clinicopathological stage, we only observed that the high expression of STK3 was associated with lymph node metastasis, suggesting the promoting role of STK3 in GC metastasis.

However, in the general concept, YAP1 phosphorylation by upstream kinases such as STK3 inhibits the translocation of YAP1 from cytoplasm into nucleus, which quenches the activation of YAP1 target oncoproteins [3, 13]. So why does STK3 depletion cause growth inhibition? Indeed, some reports indicated that YAP1 is not always an oncoprotein, and it may process more complex roles in tumorigenesis than anticipated [14–16]. Notably, overexpressing the mutant YAP1 (S127A), suppressing LATS1/2 kinases that phosphorylate S127 on YAP1, or inhibiting upstream kinases STK3/4, all could lead to growth suppression in lung cancer [15]. It suggested that the tumor-suppressive effects of STK3 may be related to the phosphorylation sites on YAP1. In addition, we even found that, from the transcriptional level, high STK3 expression was positively correlated with YAP1 signature (target genes) by GSEA (Additional file 2: Fig. S2). Our current results support that STK3 predominantly activates the non-classical pathway, Ras-MAPK, to drive GC progression. The mechanisms will be further validated by high-throughput screening in the future studies.

In light of the overexpression of STK3 in GC patients, we finally investigated the clinical associations of STK3 in GC by TCGA cohort (*n* = 375) and our in-house Hong Kong cohort (*n* = 263). Through the tissue microarray immunohistochemistry, we observed that positive STK3 was predominantly localized in the cytoplasm of the tumor tissues, while the adjacent nontumorous tissues exhibited negative expression (Fig. 3h). High STK3 expression was detected in a proportion of tumor samples, both intestinal (29.8% positive) and diffuse-types (21.2% positive) (Fig. 3i). Kaplan-Meier survival curves demonstrated that high STK3 expression was associated with worse survival in both TCGA and Hong Kong cohorts (*P* = 0.035, TCGA cohort, Fig. 3j; *P* < 0.001, Hong Kong cohort, Fig. 3k). To detailed evaluate the clinical significance of STK3 in GC, we re-analyzed its expression in intestinal-type and diffuse-type respectively, and found that abundantly expressed STK3 was associated with unfavorable clinical outcome trends in both types (Fig. 3l, m, n, and o). In addition, high expression of STK3 was correlated with poor prognosis in other cancer types as well (*P* < 0.05, Additional file 3: Fig. S3) [17], supporting the potential oncogenic role of STK3 in solid tumors. By univariate analysis, high STK3 expression was associated with lymph node metastasis (*P* = 0.047) and marginally linked with elder age (*P* = 0.058) (Additional file 4: Table S1). Multivariate analysis indicated that STK3 serves as an independent prognosis marker to predict worse survival in GC patients (Additional file 5: Table S2). So, can we employ small molecules to directly target STK3?

Indeed, XMU-MP-1, an STK3/4-specific inhibitor, has been used in some studies. It is suggested that XMU-MP-1 could augment organ repair in mice [18], supporting the suppressive role of STK3/4 in tissue regeneration. On the contrary, a recent study indicated that XMU-MP-1 slows cancerous cell proliferation, Matrigel invasion, and tumor spheroid growth in prostate cancer [6]. Very interestingly, STK3 was found frequently amplified in prostate cancer, which was concordant with our findings.

Overall, our findings fully support that STK3 serves as a potent oncogene in gastric carcinogenesis and serves as an independent prognostic biomarker in GC.

## Conclusions

Collectively, STK3 is overexpressed in a proportion of GC, and its abundance predicts unfavorable clinical outcomes. STK3 promotes cell cycle progression by activating the Ras-MAPK signaling pathway, and its depletion exerts anti-tumor effects. Our studies not only revealed the oncogenic role of STK3 kinase but also provided a therapeutic target for GC.

## Supplementary Information

**Additional file 1.**

**Additional file 2.**

**Additional file 3.**

**Additional file 4.**

**Additional file 5.**

## Data Availability

The datasets used and/or analyzed during the current study are available from the corresponding author on reasonable request.

## Abbreviations

GC: Gastric cancer
STK3: Serine/threonine kinase 3
YAP1: Yes-associated protein 1
TAZ: WW domain-containing transcription regulator protein 1
N: Normal
T: Tumor
TCGA: The cancer genome atlas
ACRG: Asian cancer research group
scRNA-seq: Single-cell RNA sequencing
GSEA: Gene set enrichment analysis
GO: Gene ontology
KEGG: Kyoto encyclopedia of genes and genomes
IHC: Immunohistochemistry
PARP: Poly (ADP-ribose) polymerase
TNM: Tumor-node-metastasis
