# Supplementary material for: STK3 promotes gastric carcinogenesis by activating Ras-MAPK mediated cell cycle progression and serves as an independent prognostic biomarker
Source: Mol Cancer. 2021 Nov 12;20:147. doi: 10.1186/s12943-021-01451-2 (PMC8588685; doi:10.1186/s12943-021-01451-2)
Supplement: Supplementary file 1 — Additional file 1. [file 12943_2021_1451_MOESM1_ESM.pdf]

Figure S1

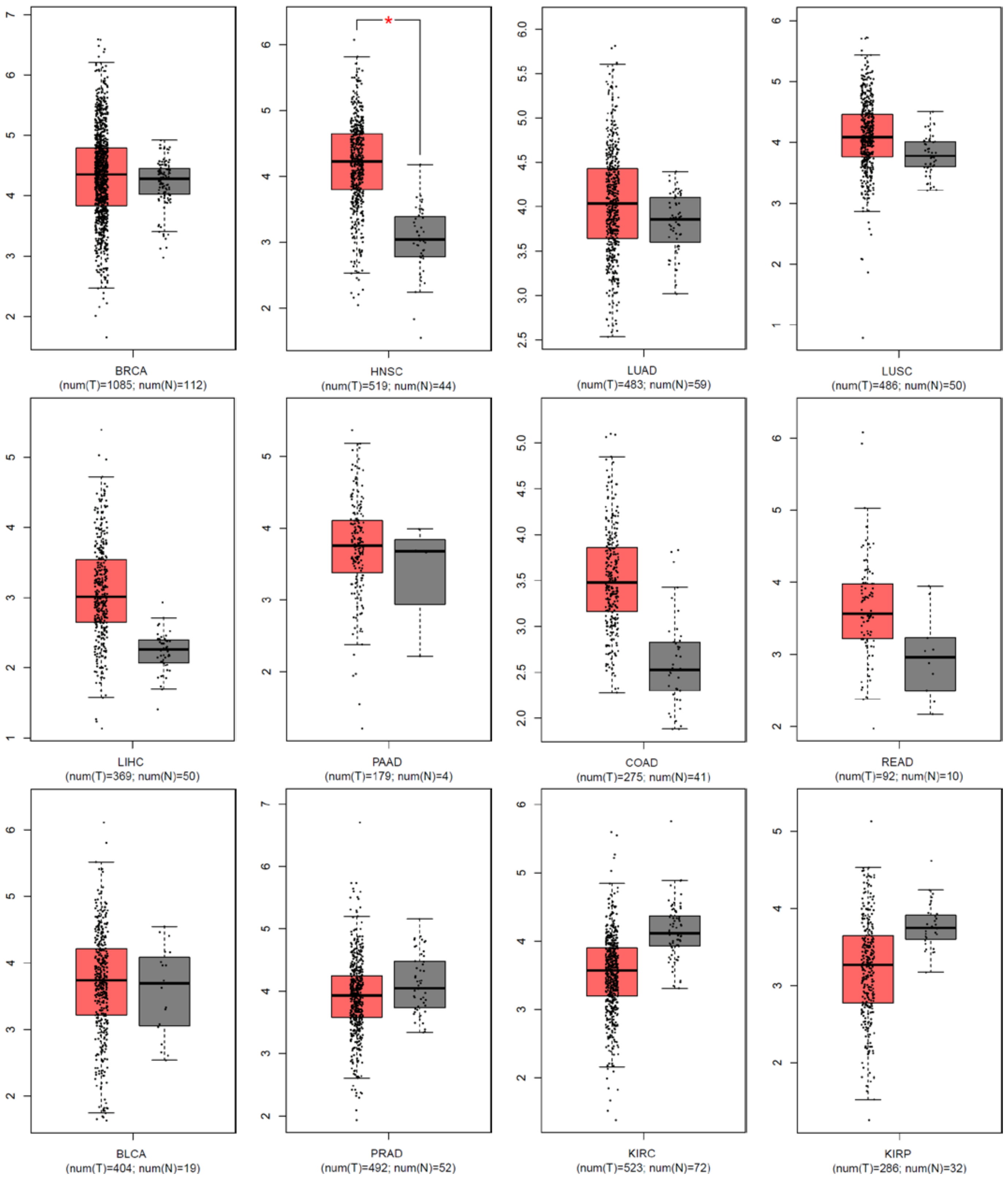

Figure S1: The expression of STK3 in multiple cancer types (expression in tumors vs in normal tissues). In most of the solid tumors, the expression in STK3 in tumor tissues demonstrates upregulated expression trend, but in prostate cancer and kidney cancer, STK3 shows decreased expression in tumor samples compared with normal. The data is generated by Gene Expression Profiling Interactive Analysis (GEPIA, http://gepia.cancer-pku.cn/). BRCA, Breast invasive carcinoma; HNSC, Head and Neck squamous cell carcinoma; LUAD, Lung adenocarcinoma; LUSC, Lung squamous cell carcinoma; LIHC, Liver hepatocellular carcinoma; PAAD, Pancreatic adenocarcinoma; COAD, Colon adenocarcinoma; READ, Rectum adenocarcinoma; BLCA, Bladder Urothelial Carcinoma; PRAD, Prostate adenocarcinoma; KIRC, Kidney renal clear cell carcinoma; KIRP, Kidney renal papillary cell carcinoma.
