# Supplementary material for: STK3 promotes gastric carcinogenesis by activating Ras-MAPK mediated cell cycle progression and serves as an independent prognostic biomarker
Source: Mol Cancer. 2021 Nov 12;20:147. doi: 10.1186/s12943-021-01451-2 (PMC8588685; doi:10.1186/s12943-021-01451-2)
Supplement: Supplementary file 2 — Additional file 2. [file 12943_2021_1451_MOESM2_ESM.pdf]

Figure S2

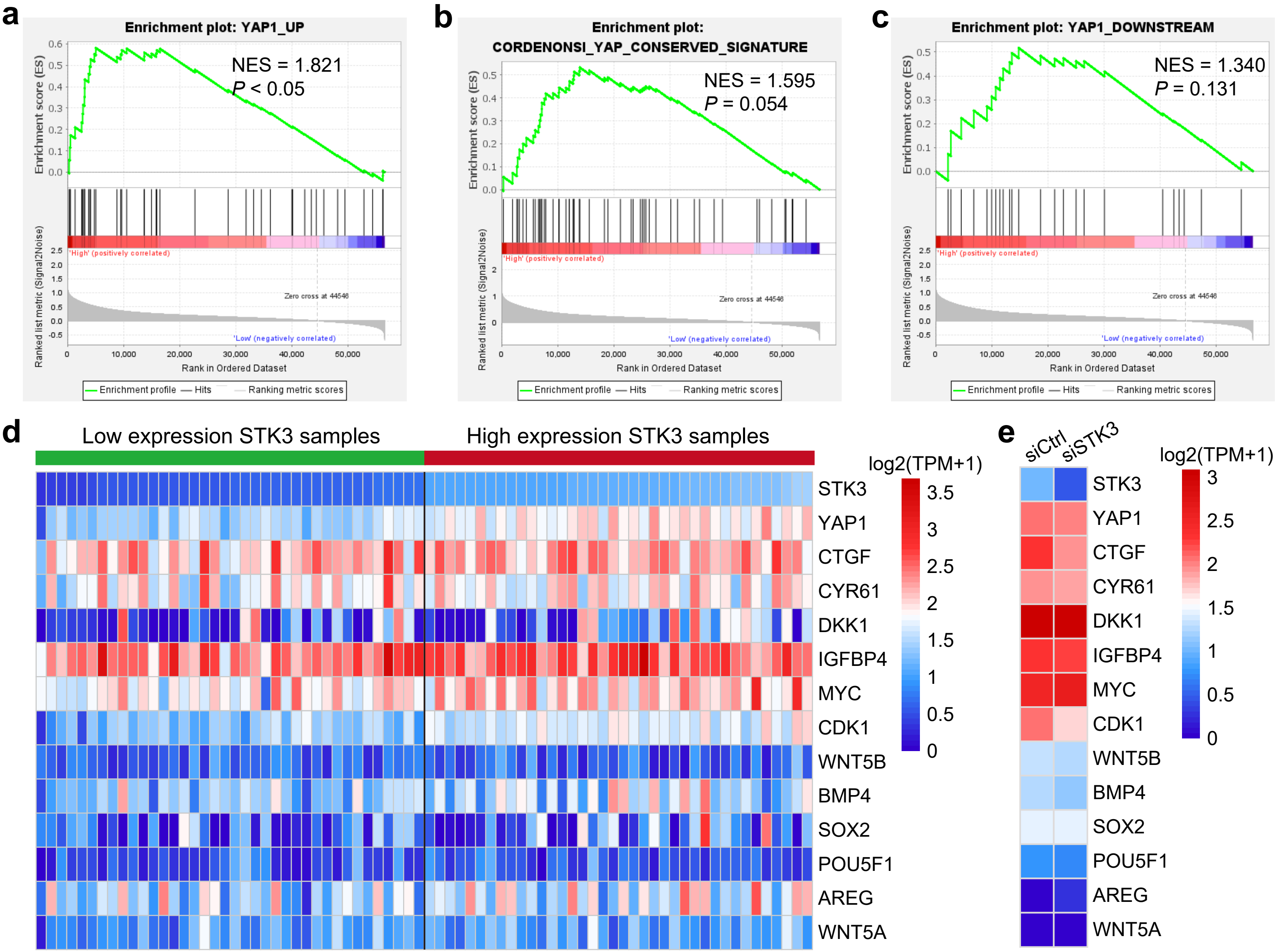

Figure S2: The expression of STK3 demonstrate positive expression with YAP1 signature. In TCGA cohort, the top 10% STK3 high expression cases demonstrate positive correlation with **a** YAP1 up, **b** YAP1 conserved signature and **c** YAP1 downstream by GSEA analysis. **d** The heatmap of STK3 expression with YAP1 signature genes in TCGA cohort. **e** Knocking STK3 decreased some YAP1 target gene expression, such as CTGF, CDK1, and WNT5B.
