# Supplementary material for: STK3 promotes gastric carcinogenesis by activating Ras-MAPK mediated cell cycle progression and serves as an independent prognostic biomarker
Source: Mol Cancer. 2021 Nov 12;20:147. doi: 10.1186/s12943-021-01451-2 (PMC8588685; doi:10.1186/s12943-021-01451-2)
Supplement: Supplementary file 3 — Additional file 3. [file 12943_2021_1451_MOESM3_ESM.pdf]

Figure S3

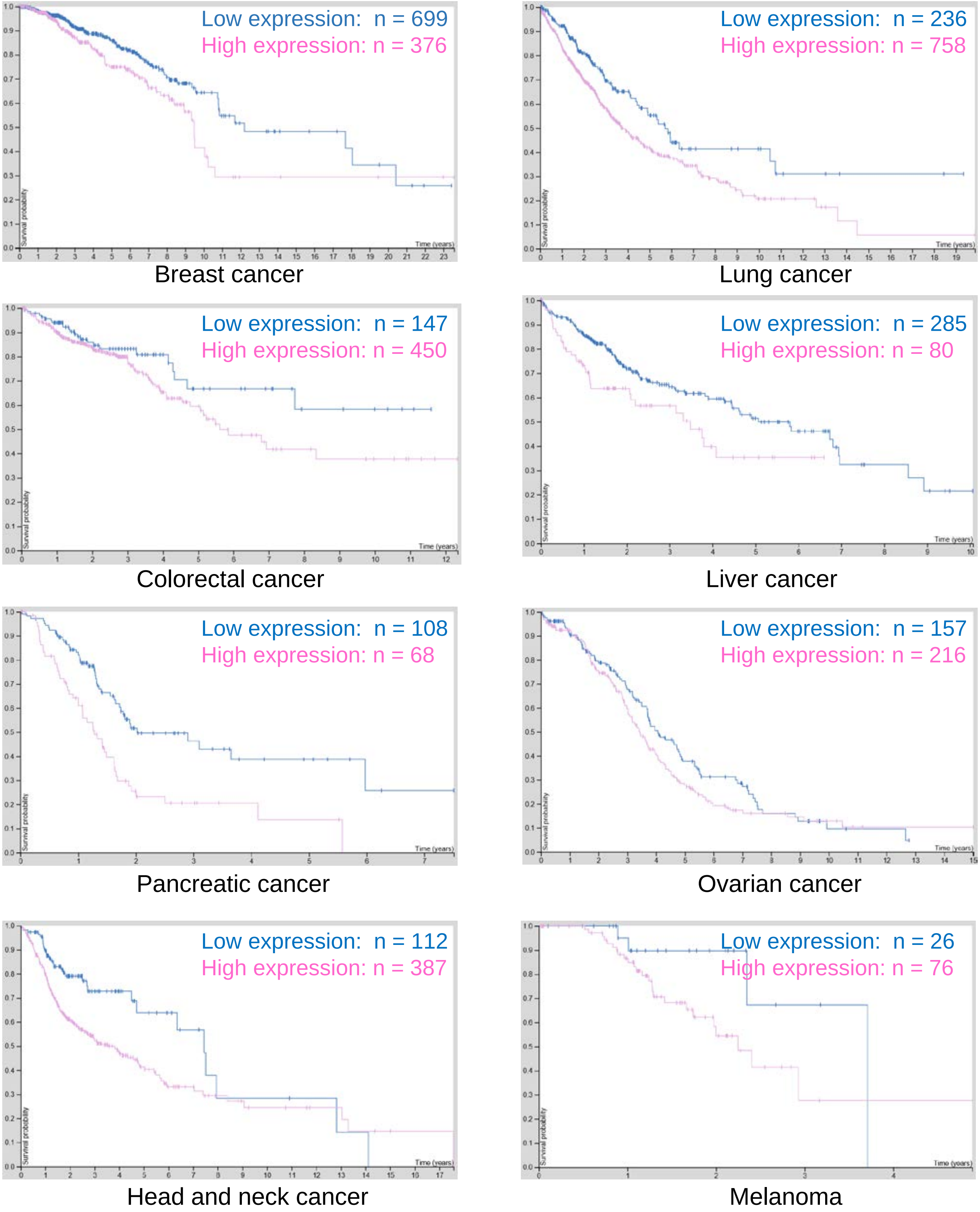

Figure S3: High expression of STK3 predicts poor clinical outcomes in multiple solid tumors. In most of the gastrointestinal cancers (colorectal, liver and pancreatic cancers), high expression of STK3 is associated with poor survival of the patients. This is also applicable in some other solid tumors, such as breast, lung, ovarian, head and neck cancer, as well as melanoma. All the *P*-value is less than 0.05 and the cohort is from TCGA and the survival curve is generated by The Human Protein Atlas (https://www.proteinatlas.org).
