# Supplementary material for: STK3 promotes gastric carcinogenesis by activating Ras-MAPK mediated cell cycle progression and serves as an independent prognostic biomarker
Source: Mol Cancer. 2021 Nov 12;20:147. doi: 10.1186/s12943-021-01451-2 (PMC8588685; doi:10.1186/s12943-021-01451-2)
Supplement: Supplementary file 4 — Additional file 4. [file 12943_2021_1451_MOESM4_ESM.docx]

| Parameters | STK3 low (n = 193) | | STK3 high (n = 70) | | *P*-value |
| --- | --- | --- | --- | --- | --- |
|  |  |  |  |  |  |
| **Age** |  |  |  |  | 0.058 |
| > 60 years | 113 | 58.5% | 50 | 71.4% |  |
| ≤ 60 years | 80 | 41.5% | 20 | 28.6% |  |
| **Sex** |  |  |  |  | 0.223 |
| Male | 134 | 69.4% | 43 | 61.4% |  |
| Female | 59 | 30.6% | 27 | 38.6% |  |
| **Tumor type** |  |  |  |  | 0.212 |
| Intestinal | 99 | 51.3% | 42 | 60.0% |  |
| Diffuse | 94 | 48.7% | 28 | 40.0% |  |
| **Tumor grade** |  |  |  |  | 0.923 |
| 1 | 9 | 4.7% | 0 | 0.0% |  |
| 2 | 66 | 34.2% | 31 | 44.3% |  |
| 3 | 118 | 61.1% | 39 | 55.7% |  |
| **TNM stage** |  |  |  |  | 0.997 |
| I | 44 | 22.8% | 14 | 20.0% |  |
| II | 22 | 11.4% | 10 | 14.3% |  |
| III | 61 | 31.6% | 24 | 34.3% |  |
| IV | 66 | 34.2% | 22 | 31.4% |  |
| **Stage (T)** |  |  |  |  | 0.916 |
| 1 | 27 | 14.0% | 9 | 12.9% |  |
| 2 | 54 | 28.0% | 20 | 28.6% |  |
| 3 | 101 | 52.3% | 39 | 55.7% |  |
| 4 | 11 | 5.7% | 2 | 2.9% |  |
| **Stage (N)** |  |  |  |  | 0.221 |
| 0 | 50 | 25.9% | 10 | 14.3% |  |
| 1 | 48 | 24.9% | 22 | 31.4% |  |
| 2 | 54 | 28.0% | 22 | 31.4% |  |
| 3 | 41 | 21.2% | 16 | 22.9% |  |
| **Stage (M)** |  |  |  |  | 0.590 |
| 0 | 163 | 84.5% | 61 | 87.1% |  |
| 1 | 30 | 15.5% | 9 | 12.9% |  |
| **Lymph node** |  |  |  |  | ***0.047*** |
| positive | 143 | 74.1% | 60 | 85.7% |  |
| negative | 50 | 25.9% | 10 | 14.3% |  |

**Table S1**. Correlation of STK3 expression by IHC in GC with other clinicopathologic features in Hong Kong cohort (n = 263, significant *P*-value in bold and Italic format). The case number and percentage counted are shown in this table.
