# Supplementary material for: STK3 promotes gastric carcinogenesis by activating Ras-MAPK mediated cell cycle progression and serves as an independent prognostic biomarker
Source: Mol Cancer. 2021 Nov 12;20:147. doi: 10.1186/s12943-021-01451-2 (PMC8588685; doi:10.1186/s12943-021-01451-2)
Supplement: Supplementary file 5 — Additional file 5. [file 12943_2021_1451_MOESM5_ESM.docx]

**Table S2**. Univariate and multivariate Cox regression analysis of the association between clinicopathologic characteristics and disease specific survival in GC patients (significant *P*-value in bold and Italic format).

| Hong Kong cohort (n = 263) | Univariate analysis | | Multivariate analysis | |
| --- | --- | --- | --- | --- |
|  | Hazard ratio (95% CI) | *P*-value | Hazard ratio (95% CI) | *P*-value |
| Sex | 1.203 (0.857-1.688) | 0.286 |  |  |
| Age (> 60 vs. ≤ 60) | 1.429 (1.012-2.018) | ***0.043*** | 2.162 (1.440-3.246) | ***0.000*** |
| Type (diffuse vs. intestinal) | 1.842 (1.324-2.563) | ***0.000*** | 1.266 (0.758-2.144) | 0.368 |
| Grade | 1.540 (1.132-2.094) | ***0.006*** | 0.948 (0.596-1.509) | 0.822 |
| TNM Stage | 2.336 (1.933-2.824) | ***0.000*** | 1.588 (1.029-2.450) | ***0.037*** |
| Stage (T) | 2.312 (1.829-2.923) | ***0.000*** | 1.516 (1.088-2.111) | ***0.014*** |
| Stage (N) | 2.011 (1.701-2.377) | ***0.000*** | 1.362 (0.985-1.882) | 0.062 |
| Stage (M) | 3.895 (2.626-5.777) | ***0.000*** | 2.673 (1.548-4.614) | ***0.000*** |
| Lymph node metastasis | 5.128 (2.888-9.104) | ***0.000*** | 0.813 (0.316-1.831) | 0.617 |
| *H. Pylori* (positive vs. negative) | 0.804 (0.571-1.132) | 0.211 |  |  |
| STK3 expression (high vs. low) | 2.229 (1.567-3.172) | ***0.000*** | 2.728 (1.826-4.076) | ***0.000*** |
